# Gallium Nitride (GaN) High-Electron-Mobility Transistors with Thick Copper Metallization Featuring a Power Density of 8.2 W/mm for Ka-Band Applications

**DOI:** 10.3390/mi11020222

**Published:** 2020-02-21

**Authors:** Y. C. Lin, S. H. Chen, P. H. Lee, K. H. Lai, T. J. Huang, Edward Y. Chang, Heng-Tung Hsu

**Affiliations:** 1 Department of Materials Science and Engineering, National Chiao Tung University, Hsinchu 300, Taiwan; nctulin@yahoo.com.tw; 2 Department of Electronics Engineering, National Chiao Tung University, Hsinchu 300, Taiwan; tommy4920@yahoo.com.tw (S.H.C.); tom2657140@gmail.com (P.H.L.); khlai.mse03@g2.nctu.edu.tw (K.H.L.); 3 International College of Semiconductor Technology, National Chiao Tung University, Hsinchu 300, Taiwan; joehuang@nctu.edu.tw (T.J.H.); edc@nctu.edu.tw (E.Y.C.)

**Keywords:** high-electron-mobility transistors, copper metallization, millimeter wave

## Abstract

Copper-metallized gallium nitride (GaN) high-electron-mobility transistors (HEMTs) using a Ti/Pt/Ti diffusion barrier layer are fabricated and characterized for Ka-band applications. With a thick copper metallization layer of 6.8 μm adopted, the device exhibited a high output power density of 8.2 W/mm and a power-added efficiency (PAE) of 26% at 38 GHz. Such superior performance is mainly attributed to the substantial reduction of the source and drain resistance of the device. In addition to improvement in the Radio Frequency (RF) performance, the successful integration of the thick copper metallization in the device technology further reduces the manufacturing cost, making it extremely promising for future fifth-generation mobile communication system applications at millimeter-wave frequencies.

## 1. Introduction

Gallium nitride (GaN) high-electron-mobility transistors (HEMTs) have become one of the most popular devices for high-frequency and high-power applications in recent years. Compared to traditional silicon devices, GaN material has several remarkable properties, such as better electron mobility at high electric field, wider energy bandgap (3.4 eV), higher breakdown electric field and higher saturation electron drift velocity [1,2,3]. Such excellent material properties have made AlGaN/GaN devices the streamline technology for high-frequency and high-power applications for next-generation wireless communication systems at millimeter-wave frequencies [4,5,6].

For the allocation of sufficient bandwidth to meet the stringent demand of ultrahigh data rates, operating at millimeter-wave frequencies has been a common practice for next-generation wireless communication networks. One of the main challenging issues is the unavoidable higher level of signal attenuation in free space as well as the losses induced in the transmission media. In that sense, device performance is strongly affected by the skin effect at high operating frequencies since the parasitic resistance tends to increase due to the limited cross-sectional area for current flow. Such parasitic resistance could possibly be minimized through thick metal deposition for interconnects at the device level.

Gold is usually selected as the interconnect material for III–V devices. However, the price of the material makes production cost inevitably high, making commercialization difficult. To address this issue, Au-free process technology was developed [7], which demonstrated CMOS-compatible AlGaN/GaN Metal-Insulator-Semiconductor HEMT (MIS-HEMT) device configuration for power electronics applications. In [8], an Au-free process was also reported in a thick metal deposition process using aluminum- and copper-based material as the interconnects. Detailed process steps overcoming the main fabrication challenges were included, and power devices with enhancement-mode and depletion-mode performances were demonstrated. In our approach, a thick copper metallization process is adopted as an alternative in the GaN device because copper has lower resistivity and higher thermal conductivity with lower cost than gold. Therefore, copper is considered a good candidate to replace gold for high-frequency device interconnection. Nevertheless, copper material suffers from the interdiffusion effect. A high-quality diffusion barrier of copper metallization is then required. Some reports showed that TaN, TiN, WNx and Pt can be used as diffusion barriers for copper metallization [9,10,11,12]. Among them, Pt material has the lowest resistivity. Therefore, a Pt diffusion barrier is adopted due to its extremely low resistivity, low electrical degradation features and better temperature stability in this work.

The objective of this study focuses on the investigation of the effect of thick copper metallization on device performance at millimeter-wave frequencies. In the following sections, device performance based on small-signal and large-signal characterization will be compared. In order to quantize the effect of the thick copper metallization, we have also extracted the corresponding parameters of the small-signal equivalent circuit for comparison purposes.

## 2. Device Fabrication

From the top to the bottom, the epitaxial layer structure of our device consists of an AlGaN barrier layer, an AlN spacer layer, the GaN channel layer, a thick GaN buffer layer and the SiC substrate. The device process can be divided into four major parts including the ohmic contact, mesa isolation, gate formation and thick copper metallization. First, the fabrication process started with ohmic contact forming. The ohmic region was defined by the mask aligner using the photoresist; then, deposition of the Ti/Al/Ni/Au multilayer was conducted by e-gun evaporation, followed by the lift-off process. The multilayer metal was then annealed at 850 °C for 30 s in an N_2_ ambient environment by a rapid thermal annealing system (RTA). The device mesa isolation was then performed, which defined the active region by lithography; then, an inductively coupled plasma (ICP) machine was used with Cl_2_ in an Ar ambient to etch the AlGaN and GaN layer for around 180 nm. For device gate formation, two-step e-beam lithography with spatial offset techniques were applied to achieve the Γ-gate structure and small gate length. A 100 nm SiNx passivation layer was deposited through plasma-enhanced chemical vapor deposition (PECVD). Then, the ditch for the gate stem was fabricated by e-beam lithography and SiNx etching by ICP. The second e-beam lithography pattern shifted 100 nm away from the previous location, which formed an overlap region. The size of the overlap region eventually determined the device gate length, which was around 90 nm in this study. To further enhance the gate controllability and improve the device transconductance, gate recess was performed. The gate metal deposition was then formed by Ni/Au metal stacks, followed by a lift-off process. Finally, a 100 nm SiNx layer was deposited with the nitride via the fabricated device pad region.

The thick copper metallization process started with triple photoresist coating using AZ5214E to reach a minimum thickness of 9 µm for a thick copper lift-off process. Then, exposure and development were carried out to define the pattern. Finally, the thick metal stacks with Ti (30 nm)/Pt (40 nm)/Ti (10 nm)/Cu (6800 nm) structure and thickness were deposited by e-gun evaporation. Ti layers were used to enhance the adhesion ability between Pt and ohmic as well as the adhesion of Cu–Pt interface in this study [13,14,15].

**Figure 1:**
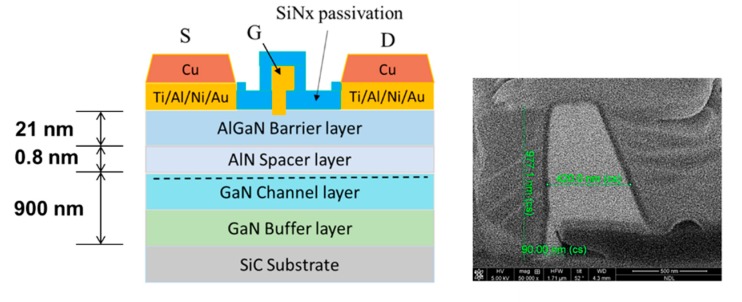
AlGaN/GaN high-electron-mobility transistors (HEMTs) epitaxial configuration and device structure with scanning electron microscope (SEM) image of the gate.

**Figure 2:**
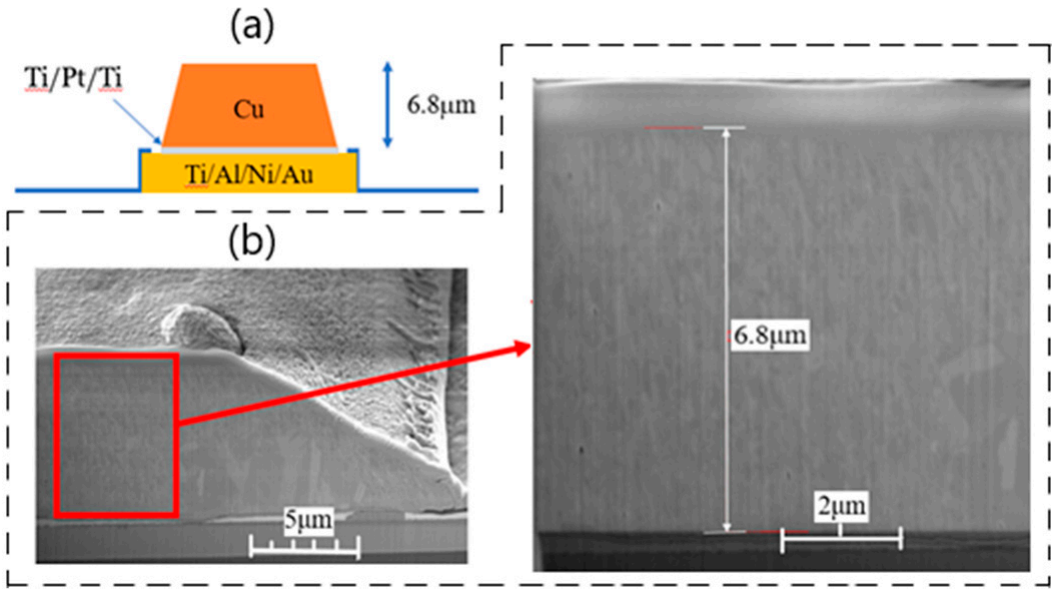
(**a**) Schematic of thick copper metallization structure. (**b**) SEM image of cross-section of thick copper metallization for GaN HEMT.

Figure 1 shows the overall epitaxial configuration and the device structure. The scanning electron microscope (SEM) image of the gate was also included in the figure. The source-to-drain distance of the device was 2 µm with the gate positioned at the center. The schematic of thick copper metallization technology and the SEM image of thick copper metallization cross-section are shown in Figure 2.

## 3. Results and Discussions

The two-port network analysis method with a small signal model was used to analyze the relationship between the drain–source current (*I_DS_*) and the transconductance (*G_m_*) versus source resistance and drain resistance. The DC and RF measurement results of devices with and without thick copper metallization were then compared. By utilizing load-pull measurement methodology, output power and power-added efficiency (PAE) characteristics could be obtained [16]. The impact of gate width on the device performance are then able to be discussed.

### 3.1. Two-Port Network Analysis

**Figure 3:**
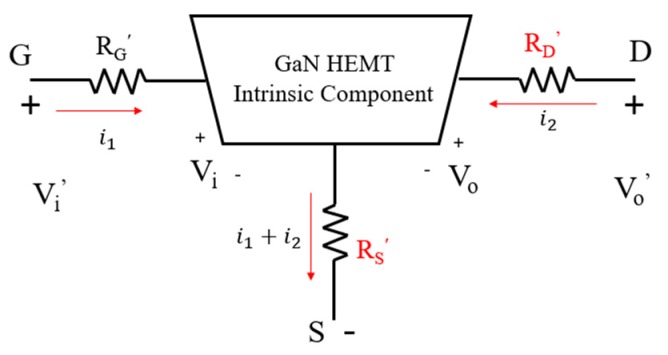
Small signal model of GaN HEMT.

With a two-port network, the small signal model of the AlGaN/GaN HEMT device can be depicted as in Figure 3 [17,18]. Utilizing the *y*-parameter analysis, the drain–source current (*I_DS_*) and the transconductance (*G_m_*) of the device can be derived as:

(1) $$I_{DS}=\frac{y_{21}v_{i}^{\prime }+y_{22}v_{o}^{\prime }}{1+y_{21}R_{S}^{\prime }+y_{22}R_{S}^{\prime }+y_{22}R_{D}^{\prime }}$$

(2) $$G_{m}=\frac{dI_{DS}}{dv_{i}^{\prime }}=\frac{y_{21}}{1+y_{21}R_{S}^{\prime }+y_{22}R_{S}^{\prime }+y_{22}R_{D}^{\prime }}$$

It is apparent from Equations (1) and (2) that the decrease of *R_S_*′ and *R_D_*′ results in the increase of *I_DS_* and *G_m_* levels.

### 3.2. DC Characteristics

**Figure 4:**
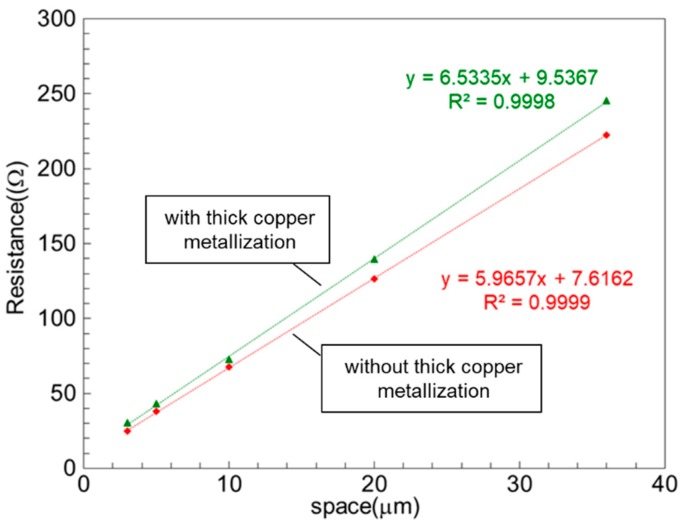
Transmission line method (TLM) measurement results before and after thick copper metallization.

The contact resistance for devices with and without thick copper metallization was measured through the transmission line method (TLM). Figure 4 shows the measurement results. The least squares regression method was adopted to find the best fit for the sets of measured data points. As expected, linear behavior was obtained, and the intersecting points with the vertical axis were extracted as the contact resistance. It was observed that the contact resistances of the samples with (green line) and without (red line) thick copper metallization were 2.5 × 10^−6^ Ω·cm^2^ and 1.7 × 10^−6^ Ω·cm^2^, respectively, leading to a difference in metal resistance of 0.96 Ω between the cases with and without thick copper metallization.

**Figure 5:**
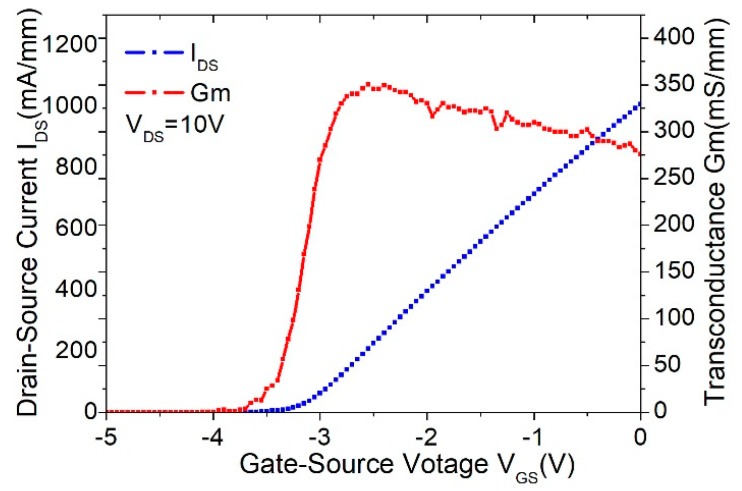
DC characteristic of the 2 × 20 μm device without thick copper metallization.

**Figure 6:**
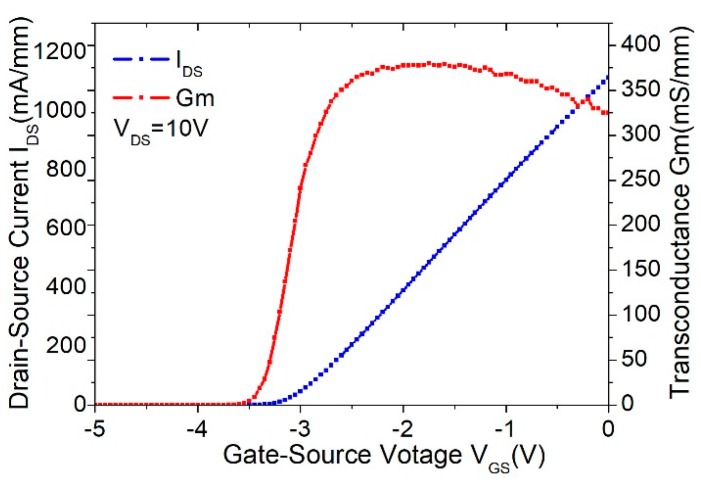
DC characteristic of the 2 × 20 μm device with thick copper metallization.

**Figure 7:**
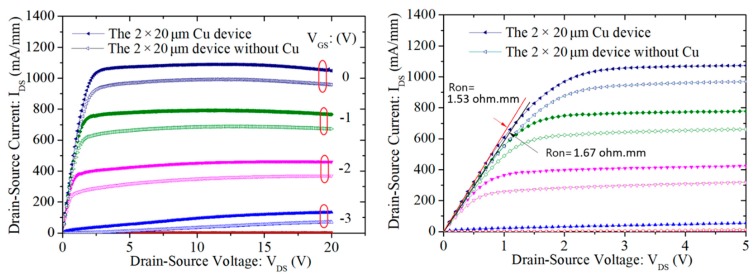
The comparison of DC I–V curves for the device with and without thick copper metallization.

To evaluate the effect of the thick copper metallization on device performances, a test device with a total gate periphery of 40 μm—which was composed of two fingers, with each finger of 20 μm in length—was fabricated. The DC characteristics, including current–voltage (*I_DS_*–*V_GS_*) relationship and the transfer curve (*G_m_*–*V_GS_*) of the device with and without thick Cu metallization, are plotted in Figure 5 and Figure 6, respectively. As observed, the device without thick copper metallization exhibited an *I_DS_* of 1010 mA/mm and a maximum *G_m_* of 350 mS/mm at *V_DS_* = 10 V. For the device with thick copper metallization at *V_DS_* = 10 V, the measured *I_DS_* was 1110 mA/mm and the maximum *G_m_* was 380 mS/mm. Such improvement in the DC characteristics was mainly attributed to the reduction in the source and drain parasitic resistance contributed by the thick copper metallization. Figure 7 shows the comparison of DC I–V curves for the device with and without thick copper metallization. The on-resistance (RON) was extracted to be 1.53 Ω·mm for the device with thick copper metallization and 1.67 Ω·mm for the device without thick copper metallization.

### 3.3. RF Characteristics

**Figure 8:**
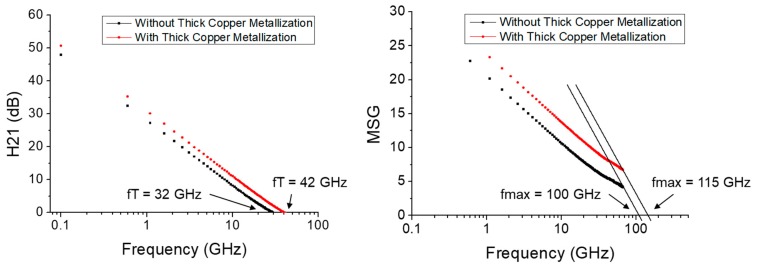
Measured small-signal performance up to 67 GHz with the extracted fT and fmax values for the devices with and without thick copper metallization.

Figure 8 shows the comparison of the measured small-signal performance for the cases with and without thick copper metallization. The measurement was performed using a vector signal analyzer in an on-wafer probing system up to 67 GHz. With the DC bias set at the maximum transconductance, the unit-current-gain cutoff frequency (fT) and the maximum oscillation frequency (fmax) were also extracted for the extrinsic device without de-embedding. As observed, the fT (fmax) of the device with thick copper metallization was 42 GHz (115 GHz) compared to that of 32 GHz (100 GHz) for the device without thick copper metallization.

**Figure 9:**
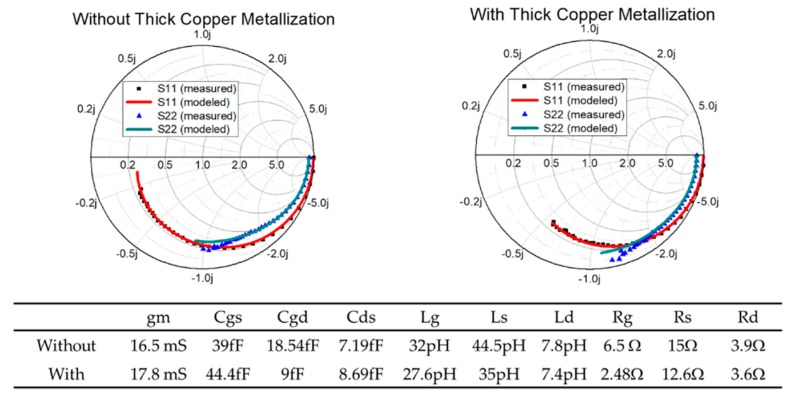
Measured and predicted S-parameters for the 2 × 20 μm device with (**right**) and without (**left**) thick copper metallization. The small-signal circuit model was extracted using the procedure defined in [19].

To further quantize the effect of the thick copper metallization, we performed the extraction of the parameters of the small-signal equivalent circuit for the devices following the same procedures outlined in [19]. All the corresponding parasitic components extrinsic to the active region of the device were extracted using both cold forward and cold pinchoff bias conditions as defined. Figure 9 shows the S-parameters measured and predicted using the small-signal equivalent circuit model. Good agreement between the measurement and prediction was obtained up to 67 GHz. The corresponding parameter values were also included for comparison. As observed, devices with thick copper metallization generally exhibited lower parasitic resistance values, which contributed to the higher fmax measured. Additionally, lower gate capacitances were extracted from the measurement for the device with thick copper metallization.

**Figure 10:**
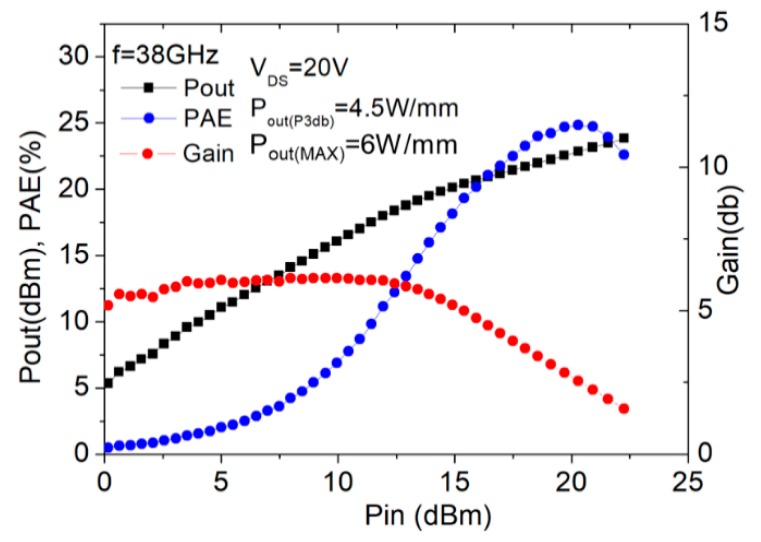
Large-signal performance of the 2 × 20 μm device without copper metallization.

**Figure 11:**
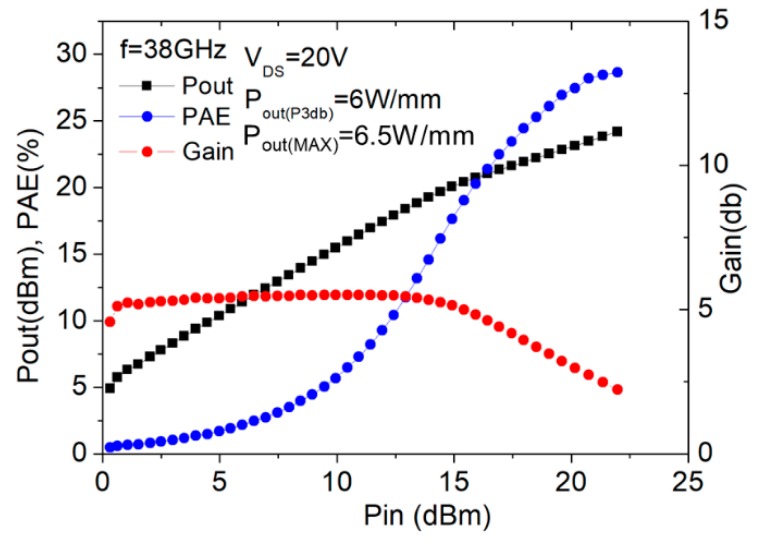
Large-signal performance of the 2 × 20 μm device with copper metallization.

On-wafer load-pull characterization (continuous mode) was also performed to investigate the power performance at 38 GHz using an automatic tuning system; the measurement results for the device without and with thick copper metallization are shown in Figure 10 and Figure 11, respectively. The output power and power-added efficiency (PAE) were compared at 3-dB gain compression with respect to the small-signal gain. With the drain bias set at 20 V, the gate bias for the device with thick copper metallization was set at −2 V and that for the one without thick copper metallization was −1.7 V. The corresponding quiescent drain current was 21 mA for the device with thick copper metallization and 19 mA for the one without thick copper metallization, with both being close to Class A operation. The measured power density and PAE for the device with thick copper metallization were 5.9 W/mm and 28.7%, compared to those of 4.5 W/mm and 24.8%, respectively, for the one without thick copper metallization.

As mentioned, for operation at millimeter-wave frequencies, the skin effect would force the current to flow on the surface of the interconnects, leading to the limitation of the effective area for current distribution, which in turn gives rise to the effective resistances at RF frequencies. Such effect could be even worse at higher frequencies since the skin depth is inversely proportional to the square root of the operating frequency. This is the major reason that the conductor loss is always dominant for planar circuits. Apparently, utilizing thick copper metallization in the device fabrication process provides a straightforward solution to such problem. From the measurement results of the 2 × 20 μm test device, it is obvious that performance improvements in the DC characteristics, the small-signal gain and the large-signal power/PAE are achieved.

### 3.4. Experimental Study of the Effect of Gate Width on the Device Performance 

**Figure 12:**
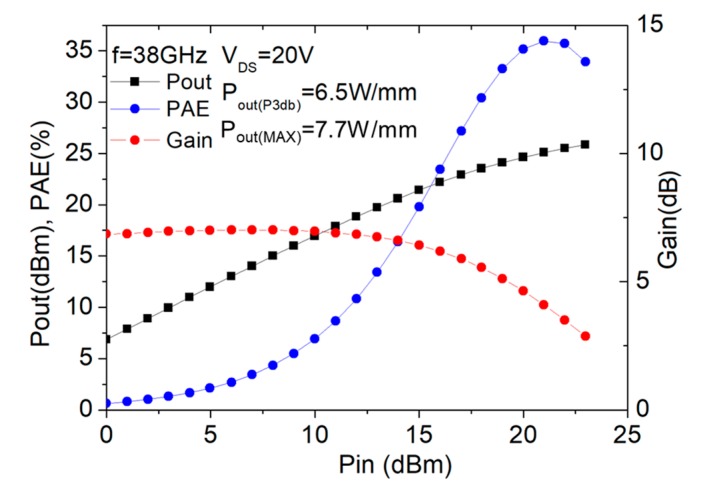
Large-signal performance of the device with gate width of 2 × 25 μm at 38 GHz.

**Figure 13:**
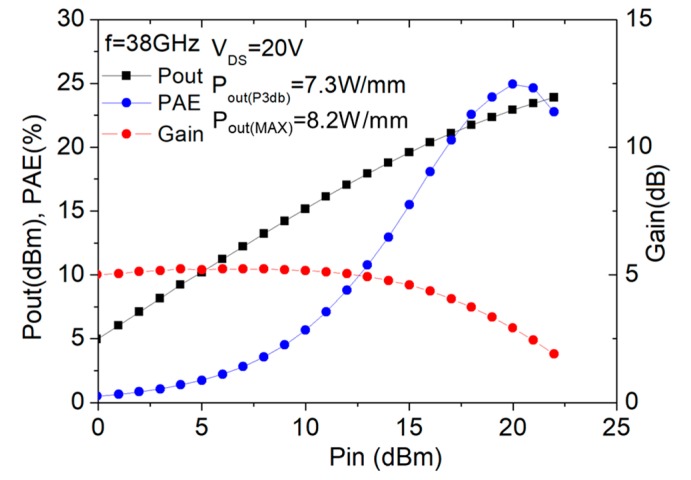
Large-signal performance of the device with gate width of 2 × 15 μm at 38 GHz.

Based on the previous conclusions, we have fabricated and characterized the devices with different gate peripheries, namely, 2 × 25 μm and 2 × 15 μm, with thick copper metallization. The corresponding results of the large-signal performance characterized using on-wafer load-pull system at 38 GHz are shown in Figure 12 and Figure 13 for the 2 × 25 μm and 2 × 15 μm devices, respectively. As shown, the device with larger gate periphery (2 × 25 μm) exhibited a power density of 7.7 W/mm at the maximum output power and the peak PAE was measured to be 36% (at the corresponding power level of 6.2 W/mm). As for the device with total gate width of 2 × 15 μm, we obtained a slightly higher power density of 8.2 W/mm at the maximum output power and the peak PAE was measured to be 26% (at the corresponding power level of 7.0 W/mm). The higher PAE achieved for the device with larger gate width of 2 × 25 μm was mainly due to the higher gain resulting from the reduction of the parasitic resistance associated with the device. Table 1 lists the performance comparisons of our devices with previously published works at the Ka band. As observed, the device exhibited the power density performance comparable to the state-of-the-art device technologies.

## 4. Conclusions

In this study, the copper metallization technique for GaN HEMT devices operating at millimeter-wave frequencies has been realized. Thick copper metallization contributes to the reduction of *R_S_*′ and *R_D_*′ and alleviates skin effect under high-frequency operation, which in turn improves the DC and RF characteristics of the devices. Experimental verifications revealed that the device with 2 × 15 μm gate width exhibited a record-high maximum output power density of 8.2 W/mm, which is the highest among the state-of-the-art published results at the Ka band. Such superior results have proven the feasibility of integrating thick copper metallization in the device process and make it promising for next-generation wireless communication system applications.

## Figures and Tables

**Table 1:** Power performance comparison of the Cu metallization with other reports.

References	Freq. (GHz)	$V_{DS}$ (V)	$L_{g}$ (nm)	Device Size (μm)	Pout @PAEmax (W/mm)	PAE (%)	$P_{out,\;max}$ (W/mm)
This Work—Device 1 with Cu metallization	38	20	90	2 × 25	6.2	36.0	7.7
This Work—Device 1 without Cu metallization	38	20	90	2 × 25	5.5	32	6.5
This Work—Device 2 with Cu metallization	38	20	90	2 × 15	7.0	26.0	8.2
This Work—Device 2 without Cu metallization	38	20	90	2 × 15	6.2	23.2	7.3
[20]	30	30	60	2 × 50	2.9	21.3	−
[21]	30	25	100	2 × 50	−	46.8	6.0
[22]	30	20	150	2 × 50	5.0	39.0	6.0
[23]	35	25	200	2 × 50	5.1	42.8	−
[24]	40	25	75	2 × 50	2.7	12.5	−
[25]	40	15	100	2 × 25	2.2	18.0	2.5
[26]	40	20	200	2 × 75	1.8	18.5	2.1
[27]	40	15	60	2 × 30	−	20.1	3.3
[28]	40	15	225	2 × 50	2.0	13.0	−
[29]	40	30	160	2 × 75	−	33	10.5
